# Treatment of scarring central airway stenosis with pirfenidone: Case report

**DOI:** 10.1097/MD.0000000000031354

**Published:** 2022-10-28

**Authors:** Xiao Li, Jinbing Pan, Haoyu Qian, Yun Ma, Bulang Gao

**Affiliations:** a Department of Respiratory and Critical Care Medicine, Henan Provincial People’s Hospital, Zhengzhou University, Zhengzhou, China.

**Keywords:** cicatrix, interventional therapy, pirfenidone, scarring central airway stenosis

## Abstract

Benign scarring central airway stenosis can be managed by high-pressure balloon dilatation, laser, surgery and stent implantation. The stenosis may have a high recurrence rate that necessitates repeated treatment. Pirfenidone (PFD) has anti-fibrosis effects and has been used in a variety of fibrosis diseases. Animal experiments suggested that PFD can prevent tracheal stenosis.

**Patient concerns:**

Patients with scarring central airway stenosis usually have chest tightness, cough and dyspnea.

**Diagnosis:**

Computed tomography scanning showed stenosis of the trachea and/or bronchus. Bronchoscopy revealed occlusion or stenosis of the trachea or bronchus.

**Interventions:**

The use of PFD in combination with other interventional management was reported to treat 2 cases of tracheobronchial stenosis after injury in this study. In the combined use of PFD and interventional management, PFD could help to alleviate tracheobronchial stenosis, prolong the time interval of bronchoscopic interventional treatment, and reduce medical costs.

**Outcomes:**

The stenosis in the trachea and/or bronchus is relieved and the patients do not have any relevant symptoms.

## 1. Introduction

Scarring central airway stenosis is a frequent complication after tracheal intubation or tracheotomy. Because of tracheal mucosa ischemia, edema, inflammation, and final fibrosis caused by compression of tube balloon in the process of tracheal intubation or tracheotomy, tracheal stenosis will occur, leading to the incidence of tracheal stenosis of 0.6% to 21%.^[1,2]^ The current treatment methods include endoscopic high-pressure balloon dilation, Laser, stenosis tracheal resection, electrocautery, cryotherapy, T-tube, and silicone stent implantation.^[3]^ However, Scarring central airway stenosis has a high risk of recurrence of about 40% to 70%^[3]^ despite these management approaches, and it is necessary to find a new anti-fibrosis drug or other effective approaches. Pirfenidone (PFD) is an anti-fibrotic drug that can reduce the degree of pulmonary fibrosis in idiopathic pulmonary fibrosis^[4]^ by inhibiting the process of transforming growth factor-*β* (TGF-*β*)-mediated fibroblast proliferation and transformation into myofibroblasts.^[5]^ Animal experiments found that PFD can reduce inflammation and fibrosis of the trachea after tracheotomy, inhibit proliferation and transformation of fibroblasts and secretion of extracellular matrix, alleviate formation of scar tissue in the larynx and trachea, and inhibit TGF-*β*l in the scar tissue.^[6–8]^ Currently, PFD has not been reported in the treatment of human trachea or bronchial scar after trauma. This study presented the initial application of PFD in the treatment of secondary scarring tracheobronchial stenosis in 2 cases.

## 2. Case report

**Figure 1.**
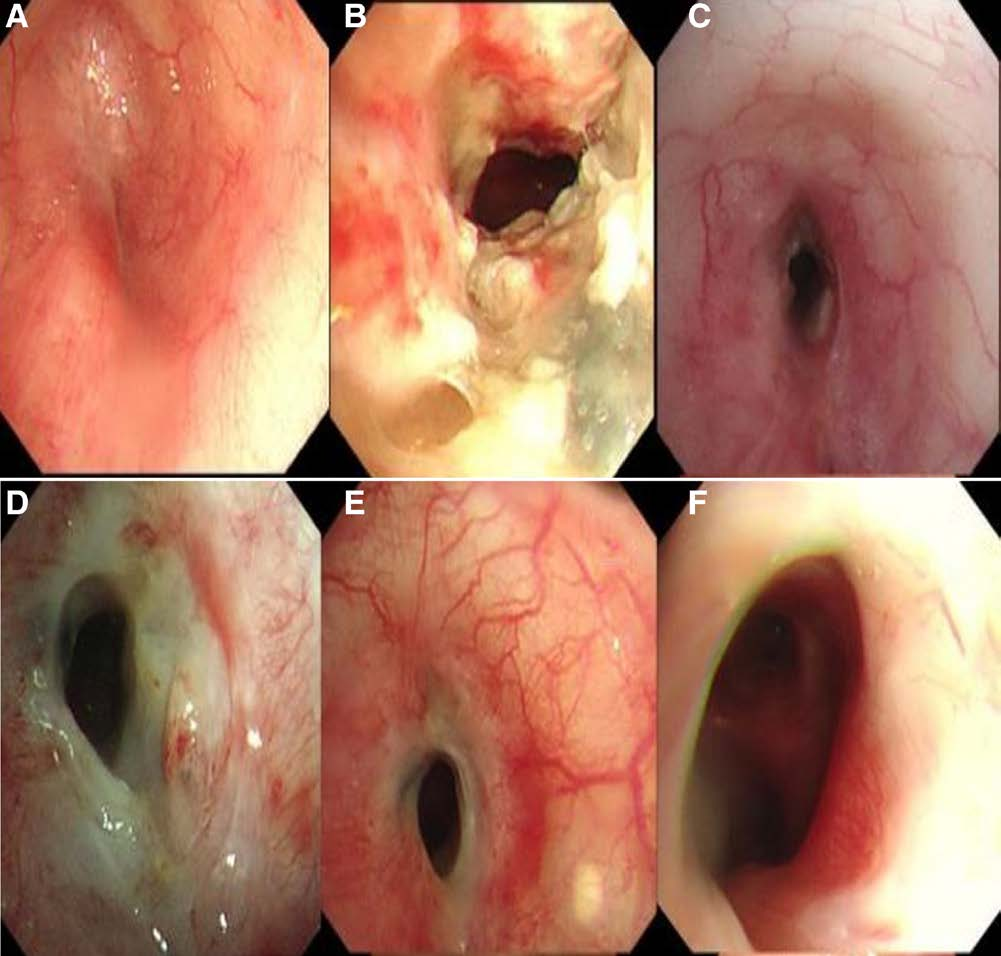
Bronchoscopy before and after PFD medication in case 1. (A) Distal occlusion of left main bronchus. (B) Necrotic tissue 7 days after recanalization of left main bronchus. (C) Narrowed lumen and granulation tissue were presented 79 days after recanalization of left main bronchus. (D) Mucosa was pale, and tough scar was formed in the narrowed segment of left main bronchus 47 days after PFD. (E) Stenotic section of left main bronchus 138 days after PFD. (F) The pale and tough mucosa was shown in the narrowed segment at the remodeling stage 265 days after PFD administration. PFD = pirfenidone.

Case 1: A 26-year-old male patient was hospitalized because of multiple body injuries in a traffic accident, including multiple rib fractures on the left side, bilateral pneumothorax, left chest collapse, left atelectasis, and left main bronchus stenosis. After chest surgery, the conditions of the patient were stable. Four months later, the patient was hospitalized again for chest tightness and cough. Physical examination demonstrated left chest collapsed, costal space narrowed, tactile tremor decreased, left lung breathing sound disappeared, right chest full, right lung breathing sound normal. Chest computed tomography (CT) showed left chest collapse, old rib fractures, left pneumothorax, pneumomediastinum, left atelectasis, and left mediastinum shift. Bronchoscopy (BF-1T260, Olympus Japan) (1st day) revealed cicatricial occlusion in the distal end of left main bronchus (Fig. 1A). After repeated treatment with cryoprobe and columnar electrotome, bronchoscopy (BF-XP260f, Olympus Japan) revealed a large amount of yellow secretion in the trachea opening and a stenosis of about 2.5 cm in length. A high-pressure balloon (6 mm × 30 mm, COOK America) was used to dilate the stenosis. After dilation, the body of the bronchoscope could be squeezed into the lumen, and a high-pressure balloon (12 mm × 55 mm, COOK America) was used to dilate further (Fig. 1B) before granulation and necrotic tissue were cleaned out. After the first bronchoscopy, repeated bronchoscopic treatment (cryotherapy, needle electrochemical scar release, high-pressure balloon dilatation and injection of triamcinolone acetonide into the narrow bronchial wall) was carried out (Fig. 1C). PFD (Continent Pharmaceuticals LTD, Beijing, China) was given orally at a dose of 200 mg 3 times a day on the 79th day. On the 47th day after medication, the medicine was stopped for 3 weeks and then continued. During the treatment, the distal lumen of the left main bronchus remained narrowed on repeated bronchoscopy (Fig. 1D), but the bronchoscope could pass smoothly. The mucosa of the narrowed bronchus was pale, but the granulation and necrotic tissues were reduced (Fig. 1E). On the 133rd day after medication, PFD was stopped. On the 260th day, repeated bronchoscopy revealed pale, tough, cicatricial stenosis. After electric knife and high-pressure balloon dilatation (10 mm × 30 mm, Boston science America), the lumen of the stenotic segment of the left main bronchus was significantly enlarged (Fig. 1F). The whole treatment process lasted 334 days with the patient being hospitalized 6 times in total.

**Figure 2.**
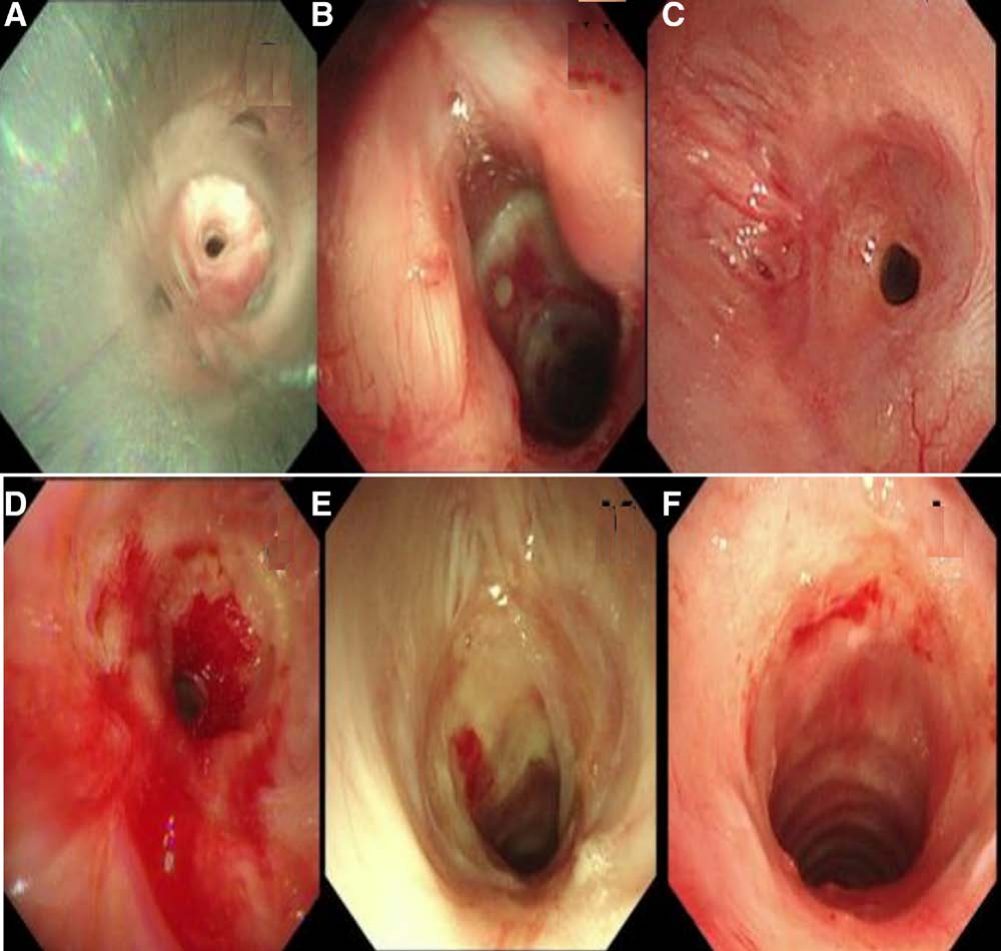
Bronchoscopy of tracheal stenosis in case 2. (A) Stenosis of the upper trachea was shown under rigid tracheoscopy. (B) Patency of the upper trachea was demonstrated after silicone stent implantation. (C) Restenosis of at the upper edge of the stent 166 days after silicone stent implantation. (D) Restenosis of the upper trachea after silicone stent removal. (E) Pale mucosa, scar formation and no significant narrowing of the lumen 24 days after PFD administration. (F) Ninety days after PFD administration, the mucous membrane of tracheal stenosis was rough and bleeding, and the lumen was not narrowed. PFD = pirfenidone.

Case 2: A 21-year-old male patient was admitted to the hospital because of cough and dyspnea with syncope twice a week (Fig. 2). Tracheotomy was performed for severe pneumonia 3 months ago. Physical examination revealed inspiratory dyspnea, respiratory rate 25 times/minute, and inspiratory rales. CT showed narrowed trachea at the level of the thyroid gland with the narrowest part of 5.2 mm and bronchiectasis in the upper lobe of the left lung. Bronchoscopy (1st day) suggested a pore-like stenosis in the trachea 1 cm away from the glottis (Fig. 2A). After local scar release, an hourglass-shaped silicone stent (18 mm-16 mm-18 mm, Dumon France) was deployed (Fig. 2B), and the patient’s symptoms were relieved. On the 166th day, the patients had dyspnea again, and bronchoscopy showed that the upper edge of the stent was narrowed to approximately 5 mm in diameter (Fig. 2C). The silicone stent was removed, and cryotherapy and drug injection with triamcinolone acetonide were administered. Later, the patient had repeated recurrent dyspnea which was treated by bronchoscope (cryotherapy, needle electrosurgical scalpel, high-pressure balloon dilatation, and triamcinolone acetonide injection) combined with PFD (Continent Pharmaceuticals LTD, Beijing, China) (orally at a dose of 200 mg 3 times a day) (Fig. 2D). Two months after the application of PFD, the patient was asymptomatic. CT revealed that the diameter of the narrowest part of the trachea was about 9.27 mm even though mild stenosis remained on bronchoscopy (Fig. 2E and F). The whole treatment process lasted 351 days with the patient being hospitalized for 6 times.

## 3. Discussion

Airway injury repair is mainly fibrous, with granulation tissue formed in the early stage and fibrous scar formed in the late stage from granulation tissue degeneration.^[9]^ Re-epithelialization provides an epithelial barrier for wound coverage, and granulation tissue forms a new scaffold for basal cell migration at the later stage of the repair process.^[10]^ The normal process of wound healing is carried out in 4 different ways: hemostasis, inflammation, proliferation, and remodeling. These stages are synchronous, chronological, overlapping, and with different types of cell-cell interactions, accompanied by a variety of cytokines, mediators, and vessels.^[11]^ Stages 1 to 3 usually last 3 weeks, whereas remodeling lasts from several weeks to years.^[10]^ Most patients will not be left with sequela, but in some patients, the accumulation of submucosal tissue may cause airway stenosis which may be further aggravated by scar contraction. For example, subglottic stenosis is the result of local fibrous repair after airway damage.^[12]^ PFD is a kind of pyridinone compound with broad-spectrum anti-fibrosis effects and can prevent and reverse formation of fibrosis and scar, which has been confirmed in the treatment of pulmonary fibrosis and other fibrosis diseases.^[13]^ PFD with the concentration of 200 to 1000 μg/mL can inhibit migration of basal cells isolated from keloid and expression of core EMT genes induced by TGF-*β*1. PFD at the dose of 400 μg/mL can significantly reduce the secretion of vimentin and fibronectin.^[14]^ Recent animal experiments suggest that PFD could significantly alleviate formation of tracheal scar in rat model of tracheal stenosis.^[6–8]^ PFD at the dose of 15 mg/kg/day can markedly reduce the degree of tracheal fibrosis and lumen stenosis after tracheotomy.^[15]^ Even though PFD has been indicated to have a therapeutic effect on airway stenosis after injury in in vitro and animal experiments, no studies have been reported on the effect of PFD in the treatment of human airway stenosis.

In our report, 2 cases with tracheobronchial stenosis treated with PFD combined with bronchoscopic management were reported. In the first case with keloid constitution, recurrent bronchial stenoses were presented after repeated bronchoscopic treatment, which was a vicious circle. However, application of PFD decreased the stenotic granulation and necrotic tissues in the left main bronchus, reduced the local mucosal congestion, and accelerated the scar formation and remodeling process, thus alleviating the degree of airway stenosis. In case 2 with tracheal stenosis caused by tracheotomy, the stenosis was relieved by the deployment of a stent, however, the stent was blocked by hyperplasia and scar tissue at the stent edge later, resulting in dyspnea. Despite removal of the blocked stent and repeated endoscopic interventional treatment, the tracheal stenosis was not relieved. Combination of endoscopic intervention and PFD medication resulted in relief of the tracheal stenosis even though mild stenosis remained. Further follow-up is still needed for observation of the treatment outcome in these 2 patients.

To sum up, PFD combined with endoscopic intervention may be effective in relieving trauma-induced tracheobronchial stenoses even though further study or follow-up is still needed for confirmation of this effect.

## Author contributions

**Conceptualization:** Jinbing Pan.

**Data curation:** Xiao Li, Haoyu Qian, Yun Ma, Bulang Gao.

**Formal analysis:** Jinbing Pan, Bulang Gao.

**Investigation:** Xiao Li, Jinbing Pan, Haoyu Qian, Yun Ma.

**Supervision:** Jinbing Pan.

**Validation:** Xiao Li, Jinbing Pan, Haoyu Qian, Yun Ma, Bulang Gao.

**Writing – original draft:** Bulang Gao.

**Writing – review & editing:** Bulang Gao.
